# Receptor-Mediated Internalization of L-Asparaginase into Tumor Cells Is Suppressed by Polyamines

**DOI:** 10.3390/ijms26146749

**Published:** 2025-07-14

**Authors:** Igor D. Zlotnikov, Alexander A. Ezhov, Elena V. Kudryashova

**Affiliations:** 1 Faculty of Chemistry, Lomonosov Moscow State University, Leninskie Gory, 1/3, 119991 Moscow, Russia; zlotnikovid@my.msu.ru; 2 Faculty of Physics, Lomonosov Moscow State University, Leninskie Gory, 1/2, 119991 Moscow, Russia

**Keywords:** polyamine transport system, targeted L-asparaginase, leukemia, polyamines, conjugates, competitive inhibitors

## Abstract

L-asparaginase (L-ASNase) remains a vital chemotherapeutic agent for acute lymphoblastic leukemia (ALL), primarily due to its mechanism of depleting circulating asparagine essential for leukemic cell proliferation. However, existing ASNases (including pegylated ones) face limitations including immunogenicity, rapid clearance, and off-target toxicities. Earlier, we have shown that the conjugation of L-ASNase with the polyamines and their copolymers results in significant enhancement of the antiproliferative activity due to accumulation in tumor cells. We suggested that this effect is probably mediated by polyamine transport system (PTS) receptors that are overexpressed in ALL cells. Here, we investigated the effect of competitive inhibitors of PTS receptors to the L-ASNase interaction with cancer cells (L5178Y, K562 and A549). L-ASNase from *Rhodospirillum rubrum* (RrA), *Erwinia carotovora* (EwA), and *Escherichia coli* (EcA) were conjugated with natural polyamines (spermine—spm, spermidine—spd, putrescine—put) and a synthetic branched polymer, polyethyleneimine 2 kDa (PEI2 ), using carbodiimide chemistry. Polyamine conjugation with L-ASNase significantly increased enzyme binding and cellular uptake, as quantified by fluorimetry and confocal microscopy. This increased cellular uptake translated into increased cytotoxicity of L-ASNase conjugates. The presence of competitive ligands to PTS receptors decreased the uptake of polyamine-conjugated enzymes-fatty acid derivatives of polyamines produced the strongest suppression. Simultaneously with this suppression, in some cases, competitive ligands to PTS significantly promoted the uptake of the native unconjugated enzymes, “equalizing” the cellular access for native vs conjugated ASNase. The screening for competing inhibitors of PTS receptor-mediated endocytosis revealed spermine and caproate/lipoate derivatives as the most potent inhibitors or antagonists, significantly reducing the cytostatic efficacy of polyamine-conjugated ASNases. The results obtained emphasize the complex, cell-type-dependent and inhibitor-specific nature of these interactions, which highlights the profound involvement of PTS in L-ASNase internalization and cytotoxic activity. These findings support the viability of polyamine conjugation as a strategy to enhance L-ASNase delivery and therapeutic efficacy by targeting the PTS.

## 1. Introduction

L-asparaginase (L-ASNase) has been a cornerstone of acute lymphoblastic leukemia (ALL) therapy for decades, owing to its ability to deplete circulating asparagine, an amino acid essential for leukemic cell proliferation [1,2,3,4,5,6,7,8]. However, current L-ASNase formulations suffer from limitations, including immunogenicity, short serum half-life, and suboptimal tumor penetration, leading to challenges in achieving sustained therapeutic efficacy and increasing the risk of relapse [9,10,11,12,13,14]. Furthermore, systemic L-asparagine depletion can result in significant off-target toxicities, impacting patient quality of life and treatment adherence. Therefore, there is a unmet need for improved L-ASNase delivery strategies that enhance tumor targeting and minimize systemic exposure [15,16,17,18,19].

Previously, we have developed a strategy to enhance the ability of L-ASNase to penetrate cancer cells by conjugating asparaginase with cationic polymers such as derivatives of cationic polymers, like chitosan, polyethyleneimine (PEI), PEG–chitosan, PEI-g-PEG, etc. [20,21,22]. The conjugation of L-ASNase with these copolymers resulted in an extension of the enzyme’s circulation time in the bloodstream, an enhancement of its accumulation in tumor tissue. As we hypothesized, it could be mediated by polyamine transport system (PTS) receptors, which are overexpressed in ALL cells [23,24,25,26,27,28,29] and are responsible for the cellular uptake of polyamines like spermine [15,20,23,28], spermidine [23,28,30], and putrescine [23,25,28,30].

Several PTS receptors show overexpression in various cancers, presenting potential therapeutic targets (Table S1) [31,32,33,34,35,36,37,38,39,40,41,42,43,44,45,46,47,48,49,50]. The table summarizes the expression and therapeutic implications of several receptors and transporters involved in the polyamine transport system (PTS) and metabolism in cancer. Research on polyamine transporters has been progressing. There is evidence that membrane transporters may mediate the transport of polyamines. For example, the transport proteins encoded by SLC22A1, SLC3A2, SLC22A16, and SLC12A8A can transfer putrescine or spermidine and spermine into the cells. SLC18B1 is a recently identified gene in the family of vesicle amine transporters. This protein is responsible for the storage and release of polyamine vesicles and functions as a vesicle polyamine transporter.

Recent studies have shown that ATP13A2, a late endolysosomal transport protein, is involved in lysosomal polyamine transfer [39]. The increased expression of ATP13A4 (breast cancer), ATP13A3 (pancreatic and neuroblastoma cancers), and SLC3A2/SLC7A5 (LAT1) in various cancers suggest their potential as therapeutic targets through the inhibition of polyamine transport. Glypican-1 (GPC-1) overexpression can act as a polyamine transporter, promoting cancer cell proliferation and metastasis, and may influence polyamine uptake. Overall, Table S1 highlights the complex interplay between polyamine transport, metabolism, and cancer progression, suggesting multiple potential therapeutic avenues targeting these processes.

Polyamines and their derivatives, including alkylated or elongated analogs, show promise as therapeutic agents targeting cancer cells, particularly in breast cancer treatment [49,51]. These analogs deplete polyamine pools, mimicking the effects of high natural polyamine concentrations, thereby downregulating biosynthetic enzymes (ODC and SAMDC) and upregulating catabolic enzymes (SSAT), ultimately inhibiting cellular proliferation [52,53,54,55]. The employment of polyamines as ligands for the targeted delivery of anti-cancer agents, such as L-asparaginase in nanoparticle, micellar, or liposomal form, or in gels, represents a promising approach [21].

In addition to the specific polyamine transporter proteins/channels/receptors (such as ATP13A3 or SLCs in Table S1), which directly bind and facilitate polyamine movement across the membrane, caveolae ensure a mechanism of endocytosis. Caveolae, small flask-like indentations of the plasma membrane, play also involved in polyamine uptake, especially for spermidine and spermine [56,57,58,59]. Other endocytic routes, such as clathrin-mediated endocytosis [57,60,61,62], clathrin-independent endocytosis [59], and micropinocytosis [63,64,65,66], also can contribute to polyamine transport, particularly for putrescine. These various endocytic pathways, distinct from specific protein channels/receptors, represent broader cellular internalization systems that can work in conjunction with or independently of dedicated transporters. The importance of these pathways can vary depending on the cell type and specific polyamine involved (Table S1). Transporters and channels, while not yet fully identified, are likely part of the mechanism by which polyamines cross cell membranes. Organic cation transporters (OCTs) [67,68] and members of the SLC transporter family [69,70] are potential candidates.

In order to optimize the delivery of L-asparaginase, a number of approaches can be implemented:

- The conjugation of L-ASNase with polyamines such as spermine, spermidine, or putrescine can enhance its uptake via the PTS [20,71,72].
- Encapsulating L-ASNase within nanoparticles coated with polyamines or their analogs to improve its targeted delivery to tumor cells [15].
- Inhibiting the PTS could suppress cancer cell growth and proliferation by blocking polyamine uptake [73,74,75] through the use of polyamine derivatives with fatty tails (propionate, laurate, caproate).

We propose an approach based on the upregulation of PTS expression in cancerous cells. We effectively applied this as a «Trojan horse» strategy to deliver L-ASNase into the cells and demonstrated the efficacy of polyamine-conjugated formulations of asparaginase in terms of enhanced cytostatic activity [15,20]. We hypothesized that this enhanced efficacy is due to their PTS-mediated cellular entry. 

This work aims to reveal the PTS’s role in our L-ASNase uptake. We investigate how PTS competitive inhibitors (compounds that could compete for or block PTS receptors) affect L-ASNase activity, thus confirming PTS-mediated uptake. This targeted delivery strategy is expected to improve therapeutic efficacy while potentially minimizing off-target effects.

## 2. Results and Discussion

### 2.1. Article Structure

The rationale for conjugating L-ASNases with polyamines like spermine, spermidine, putrescine, and polyethyleneimine (PEI) stems from the overexpression of PTS in many cancer cells. The promyelocytic leukemia cells of humans exhibit a remarkable affinity for putrescine, spermidine, and spermine, which is between 10 and 200 times higher than that exhibited by normal leucocytes in humans [76]. These polyamines, conjugated with L-ASNase, were studied to optimize the composition in order to achieve maximum uptake through PTS-mediated mechanisms, ultimately leading to enhanced anti-cancer activity.

To make sure that the uptake of conjugates by tumor cells is mediated by PTS, we study the impact of competitive inhibitors on the permeation and cytostatic action of enzyme conjugates.

The cytotoxic activity of the conjugates was evaluated in vitro against relevant cancer cell lines, examining the impact of different polyamines and their fatty acid derivatives on this activity. The study delved into the specific roles of the selected polyamines and their fatty acid derivatives, analyzing their influence on PTS activity, membrane permeability, and overall conjugate efficacy. Fourier-transform infrared (FTIR) spectroscopy was employed to investigate the intricate interactions between cells and both native L-ASNase and the conjugated forms, providing insights into cellular uptake mechanisms and specific interactions with cellular components.

### 2.2. The Synthesis and Characterization of Polyamine-Conjugated L-ASNase Formulations

The study involved the synthesis of conjugates of L-ASNase enzymes obtained from three different microbial sources: *Rhodospirillum rubrum* (RrA), *Erwinia carotovora* (EwA), and *Escherichia coli* (EcA). These enzymes, possessing distinct physico-chemical properties (as partially summarized in Table 1 regarding quaternary structure, monomer molecular weight, pH/temperature optima, activity, and Michaelis constant *K*_M_), were conjugated with different amine-containing molecules. The conjugating agents included natural oligoamines—spermine (spm), spermidine (spd), putrescine (put)—and a synthetic branched polymer, polyethyleneimine (PEI), with an average molecular weight of 2 kDa.

**Figure 1:**
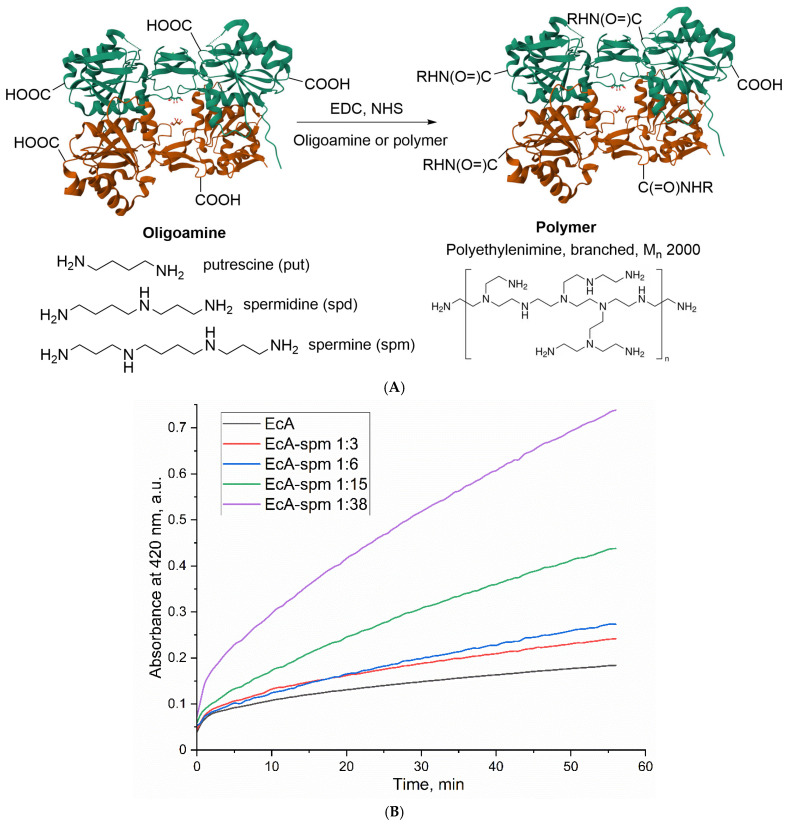
The scheme of synthesis of L-ASNase conjugates with polyamines (**A**). Kinetic curves of the spectrophotometric titration of an amino group composed of L-ASNase and their conjugates with oligoamines using TNBS reagent. Absorbance was measured at 420 nm. C(L-ASNase) = 10 µg/mL. Sodium borate buffer (20 mM, pH 9.2) (**B**).

The conjugation was achieved using the well-established carbodiimide coupling method, schematically depicted in Figure 1A. This strategy relies on the activation of surface-exposed carboxyl groups (–COOH) on the L-ASNase protein using 1-Ethyl-3-(3-dimethylaminopropyl)carbodiimide (EDC) in the presence of N-hydroxysuccinimide (NHS). EDC facilitates the formation of a reactive O-acylisourea intermediate, which is subsequently converted into a more stable, amine-reactive NHS-ester by NHS. These activated carboxyl groups then undergo nucleophilic attack by the primary amine groups present in the oligoamines or PEI. This results in the formation of stable amide bonds, covalently linking the polyamine or PEI moiety to the enzyme surface.

To explore the impact of the modification degree on the enzyme properties, conjugates were prepared using varying molar ratios of the amine component (spm, spd, put, or PEI) to the L-ASNase tetramer. The extent of the modification of asparaginases by polyamines was determined through the process of titrating amino groups using the TNBS reagent. Figure 1B displays the titration curves of primary amino groups in native L-ASNase and its conjugates with increasing amounts of oligoamines attached to the enzyme. A significant increase in the number of titrated amino groups is observed for the conjugates compared to the native enzyme, indicating the successful conjugation of the oligoamines/polymers. This increase is quantitatively presented in Table S2, which reports the number of titrated amino groups per L-ASNase tetramer at increasing molar excesses of each oligoamine or polymer. The data reveal a dose-dependent increase in amino group modification upon increasing amounts of oligoamines/polymers. For instance, the number of titrated amino groups for EcA increases from 88 (for native EcA) to 126–151 (for ASP conjugates with polyamines). The specific ranges of molar ratios for each enzyme type and amine are detailed in Table 1 and Table S2. Therefore, TNBS titration data confirm the successful and efficient conjugation of these molecules onto the L-ASNase surface.

**Figure 2:**
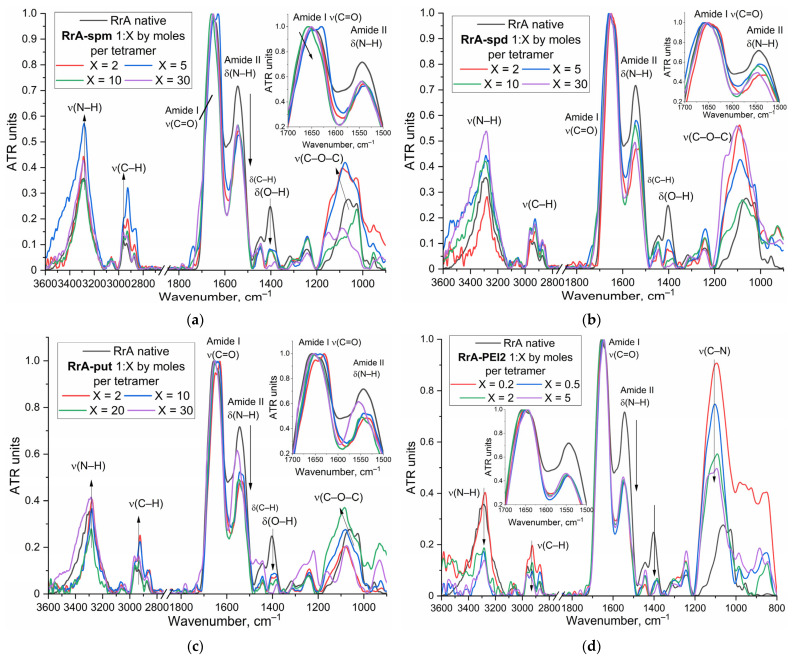
Normalized FTIR spectra of conjugates of L-ASNase with (**a**) spm, (**b**) spd, (**c**) put, and (**d**) PEI2 in different molar ratios of polyamine: protein tetramer (used in synthesis).

Following synthesis, the successful formation of the L-ASNase-polyamine conjugates was confirmed using FTIR spectroscopy, with representative spectra shown in Figure 2a–d for native RrA and its conjugates. Normalized FTIR spectra of the conjugates exhibit distinct differences compared to the spectrum of native RrA, providing clear evidence of chemical modification. The extent of these spectral changes generally correlates with the increasing molar ratio (X) of the attached molecule, indicating a dose-dependent modification (molar ratios of polyamine: protein tetramer used in synthesis). The amide I band (around 1650 cm^−1^, mainly C=O stretch) and amide II band (around 1550 cm^−1^, N-H bend coupled with C-N stretch) are characteristic protein backbone signals. Upon conjugation, a noticeable increase in the relative intensity of the amide II band normalized on amide I band (the inserts in each Figure 2 provide a enlarged view of this crucial region) is observed across all conjugate types (spm, spd, put, PEI2). The intensity of the 3000–2800 cm^−1^ bands corresponding to aliphatic C-H stretching vibrations (v(C-H)) increases in the conjugates compared to native RrA due to the high density of the backbones of methylene-group polyamines. Changes in the broad band (v(N-H), v(O-H) in 3600–3000 cm^−1^) in this region are observed, likely reflecting the contribution of the added amine (N-H) groups from the polyamines/PEI and alterations in the hydrogen-bonding network within the modified protein. Finally, changes in bands associated with O-H bending (δ(O-H) around 1400 cm^−1^) and various C-O and C-N stretching modes around 1100 cm^−1^) further support the structural changes resulting from conjugation. The conjugation reaction yield of L-asparaginase with polyamines exceeded 90% (assessed by the C-O band at 1100 cm^−1^), confirming that the true ratio closely approximates the initial input values presented in Table 1.

### 2.3. Biocatalytic Parameters of the Polyamine-Conjugated L-ASNases

**Figure 3:**
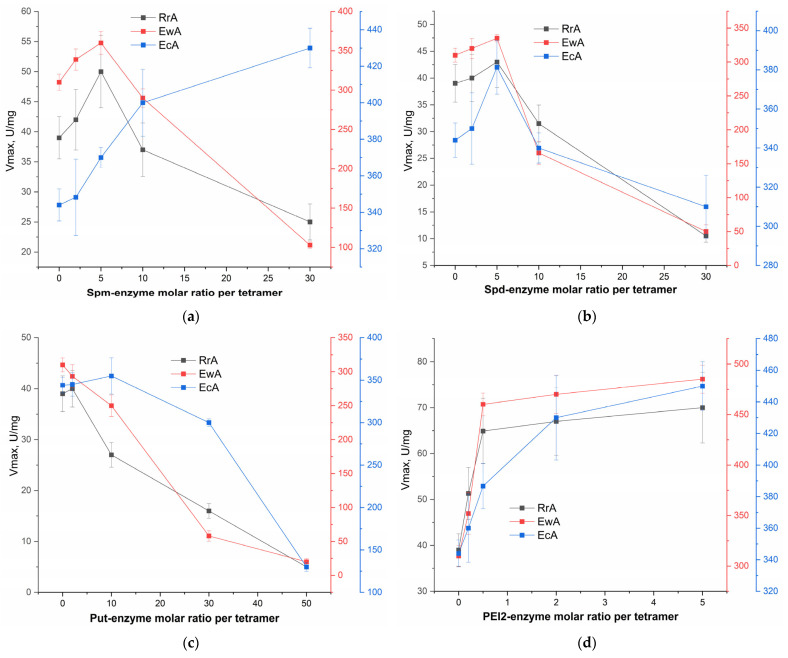
The kinetic parameters (Vmax, U/mg) of hydrolysis reactions of 1 mM fluorescent substrate Asp-AMC catalyzed by native L-ASNase and its conjugates with (**a**) spm, (**b**) spd, (**c**) put, and (**d**) PEI2 in different molar ratios of polyamine: protein tetramer (used in synthesis). PBS (0.01 M pH = 7.4). T = 37 °C. The average values are presented, indicating the standard deviation for three independent measurements.

Our primary objective in this study was to determine the optimal parameters for L-asparaginase modification and the resulting degrees of polyamine conjugation. This is particularly crucial because L-ASNase is highly sensitive to the introduction of positive charges, especially concerning its catalytic activity [21]. To evaluate the effect of conjugation, the catalytic parameters (Vmax) of the synthesized L-ASNase conjugates with polyamines were determined using a previously validated fluorometric assay [22]. This robust method allowed for the precise measurement of the enzyme’s activity by monitoring the hydrolysis of the fluorescent substrate, Asp-AMC. Figure 3 presents the Vmax values obtained from the hydrolysis reactions of 1 mM Asp-AMC catalyzed by native L-ASNase and its conjugates with (a) spermine (spm), (b) spermidine (spd), (c) putrescine (put), and (d) PEI2, at various molar ratios of polyamine to L-ASNase. A comparison of the catalytic efficiency between the native enzyme and its modified counterparts elucidates the significant impact of polyamine conjugation on L-ASNase activity.

#### 2.3.1. Effect of Spermine Modification on the Catalytic Activity of L-ASNase

Modification with spm (Figure 3a) showed the optimal range of modification observed for RrA and EwA enzymes. RrA activity increased by 21% to 50 U/mg at the spm–enzyme ratio of 1:5, and EwA—by 16%, to 360 U/mg. However, at high ratios (1:10 and 1:30), activity decreased, which indicates a negative effect of excess charge or structural changes. EcA, on the other hand, showed an increase in activity with an increase in the spm ratio, peaking at 1: 30 (430 U/mg, +26%).

#### 2.3.2. Effect of Spermidine Modification on the Catalytic Activity of L-ASNase

Spd (Figure 3b) in low and moderate concentrations showed much smaller effects than the spermine effect on RrA and EwA, with a maximum increase in activity at a ratio of 1:5. However, high ratios (1:10 and 1:30) dramatically reduced the activity of both enzymes. The EcA also showed an increase in activity at 1:5 (381 U/mg, +11%), but a further increase in the ratio resulted in a decrease in activity.

#### 2.3.3. Effect of Putrescine Modification on the Catalytic Activity of L-ASNase

Putrescine (Figure 3c) showed a mostly negative effect on the activity of all enzymes. Even at low ratios of 1:2, the activity of RrA and EcA remained virtually unchanged, while EwA decreased slightly. At high ratios (1:30 and higher), the activity of all enzymes significantly decreased: RrA—up to 13% (5 U/mg), EwA—up to 6% (20 U/mg), and EcA—up to 38% (130 U/mg).

#### 2.3.4. Effect of PEI2 Modification on the Catalytic Activity of L-ASNase

PEI2 showed a significant improvement in the activity of all enzymes at moderate concentrations (1:2 and 1:5). RrA increased activity by 80% (up to 70 U/mg), EwA by 56% (up to 485 U/mg), and EcA by 31% (up to 450 U/mg). However, at high concentrations of (1:5), the effect plateaus, which indicates the presence of an optimal range of modification.

#### 2.3.5. Comparative Analysis of Oligoamine and PEI2 Modification on L-ASNase Activity

The results obtained reveal distinct trends based on the polyamine type and degree of modification, highlighting the influence of chain length and charge density on enzyme activity. Supplementary Figure S1 provides a detailed structural representation of RrA after modification with spermines. This visualization clearly illustrates the sites of polyamine attachment, suggesting a distribution that could influence the enzyme’s surface charge and overall conformation. Such structural insights are crucial for understanding the observed changes in RrA’s activity, stability, or cellular interactions following polyamine conjugation. Polyamines (PEI2, spm, and spd), with their longer chains and higher charge densities compared to putrescine, generally showed a modest potential for activity enhancement at low to moderate modification ratios. Spm at a 1:5 ratio led to the most significant improvements, particularly for RrA and EwA. Spd also showed some benefits at a 1:5 ratio, especially for EcA. However, higher modification ratios with both spermine and spermidine consistently led to decreased activity, suggesting that excessive positive charge and/or steric hindrance negatively impacted enzyme function. Putrescine, with its shorter chain and lower charge density, showed primarily detrimental effects across all enzymes and all of modification ratios tested. This suggests that a certain minimum chain length and charge density are required for beneficial effects, but exceeding the optimal level becomes counterproductive.

For different enzymes, there are optimal modification levels at which maximum activity is achieved. Excess charge or structural changes can reduce the activity of the L-ASNase. Firstly, the introduction of a high density of positively charged polyamines can induce strong electrostatic repulsion and steric hindrance, leading to the disruption of the enzyme’s crucial tertiary structure and potentially altering or occluding the active site (Figure S1). Secondly, given L-asparaginase’s oligomeric composition, excessive modification can perturb critical inter-subunit contacts, potentially causing dissociation into less active subunits or impaired assembly. Understanding how polyamine conjugation affects cellular permeability and intracellular targeting is crucial to fully appreciating the observed enhancement in cytotoxicity.

### 2.4. The Visualization of the Interaction Between Polyamines and Lymphoma Cells

**Figure 4:**
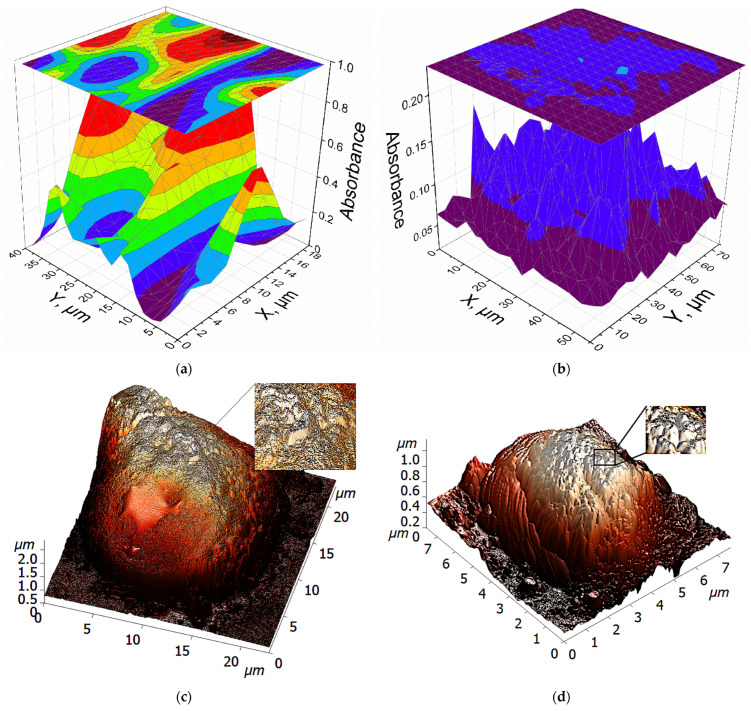
FTIR maps of the integral absorbance of (**a**) spm-eosin and (**b**) PEI2-eosin in the range 1500–1350 cm^−1^ used for labeling PTS receptors on L5178Y cells, showing clustering of polyamine receptors. The scanning step was 1 µm. Eosin was necessary for the visual identification of cells. (**c**) AFM image of L5178Y cells after treatment with PEI2. (**d**) AFM image of L5178Y cells without treatment.

To assess the quantity and localization of PTS receptors on cancer cells, we aim to elucidate the specific interactions between different polyamines and designated receptors using Fourier-transform infrared (FTIR) microscopy and atomic force microscopy (AFM) techniques. This analytical approach will provide evidence for the presence and clustering of receptors or binding sites on the cell surface that are specific for polyamines, enabling us to accurately map their distribution. Figure 4 illustrates complementary data acquired from the two distinct techniques (FTIR and AFM microscopies) employed to investigate the interactions of polyamine analogs and related molecules with the surface of L5178Y cells, specifically highlighting the PTS receptors.

FTIR chemical maps are displayed as 3D plots (Figure 4a,b), where the X and Y axes represent the spatial coordinates on the cell surface (scanned area), while the *Z*-axis and color scale represent the integrated infrared absorbance within the spectral range of 1500–1350 cm^−1^, corresponding to the eosin label, which is conjugated to polyamine ligands targeting cell-surface components. Higher absorbance (warmer colors: red, yellow) indicates a higher concentration of the eosin-labeled polyamine ligand in the specific location.

The **spm** map reveals a heterogeneous distribution, with distinct areas (clusters or domains) exhibiting significantly higher absorbance than surrounding regions (rather large regions corresponding to cells of the order of 10 microns in size). This suggests that the binding sites for spermine, presumably PTS receptors, are not uniformly distributed across the cell membrane but are localized or clustered in specific domains.

The non-uniform distribution of **PEI2**, characterized by localized regions of high concentration of the polyamine ligand (high absorbance) and individual small visible maxima (1–2 microns), is consistent with its polymeric nature and reflects multipoint interactions with the cell surface. PEI2 has a lower ability to bind to PTS receptors compared to spermine, since it is not a specific ligand. A semi-quantitative estimate gives a PEI2 binding value of 10–15%, although spermine binds by 70–80%.

Thus, FTIR chemical mapping successfully visualizes the non-uniform, patchy distribution of binding sites for specific ligand spermine and to a lesser extent less specific ligand PEI on the L5178Y cell surface, suggesting the presence of specialized membrane domains or receptor clusters associated with the polyamine transport system.

AFM technique provides complementary high-resolution topographical information about the cell-surface structure. Figure 4c shows the surface of an L5178Y cell before treatment and Figure 4d shows the surface of an L5178Y cell after treatment with PEI2. The visible surface texture and morphology of L5178Y cells, potentially showing aggregated domains as evidence of receptor clustering within the membrane of L5178Y cells.

### 2.5. Synthesis and Characterization of Polyamine Derivatives as Potential PTS Inhibitors or Membrane Permeability Enhancers

We investigated the process of L-ASNase conjugate internalization in the presence of competitors for the polyamine transport system—oligoamines and their derivatives, which can be considered as PTS effectors. Original polyamines like spermine, spermidine, and putrescine are essential for various cellular processes, including cell growth, differentiation, and nucleic acid packaging [26,27,28]. The modification of these polyamines by fatty acids or other functional groups, such as cyclopropyl or short alkyl residues [49], can significantly modify their interaction with biological membranes and PTS receptors. This alteration can impact their potential use as therapeutic agents or delivery vectors. These modifications can impact charge distribution, lipophilicity, and interaction (inhibition or activating) with PTS or membrane permeability [49,77].

**Figure 5:**
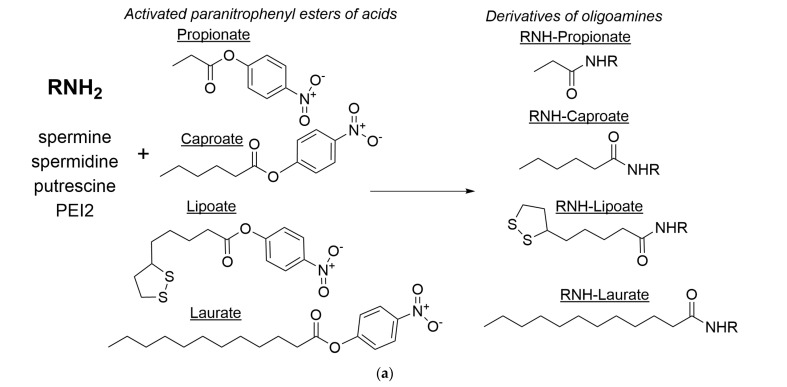

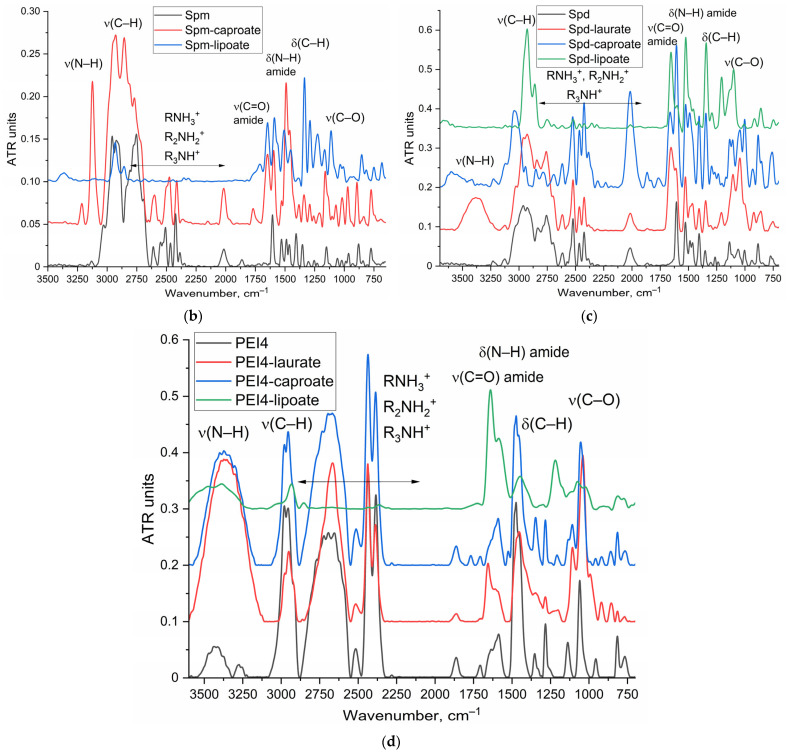
(**a**) The scheme of synthesis of polyamine derivatives as potential PTS inhibitors or membrane permeability enhancers. FTIR spectra of these dry compounds: (**b**) spm derivatives; (**c**) spd derivatives; (**d**) PEI derivatives. The arrow shows the peak range of charged amines.

Figure 5a outlines the synthesis of polyamine derivatives designed as potential PTS inhibitors or membrane permeability enhancers. Spermine, spermidine, putrescine, and PEI2 serve as the base polyamines. The reaction employs activated para-nitrophenyl esters of fatty acids: propionic (propionate), caproic (caproate), lipoic (lipoate), and lauric (laurate) acids. These activated esters facilitate amide bond formation due to the excellent leaving-group properties of para-nitrophenolate, promoting nucleophilic attack by the polyamine’s amino group on the ester’s carbonyl carbon. The reaction between the polyamine amino groups and the activated fatty acid esters results in the formation of an amide bond (-CO-NH-). An analysis of the FTIR spectra presented in Figure 5b–d confirms the successful formation of amide bonds as a result of the reaction of polyamines with activated fatty acid esters. The conjugation reaction yield of polyamines with acid residues exceeded 80% (assessed by the C-N band at 1020 cm^−1^ and C–H band at 3000–2800 cm^−1^), confirming that the true ratio closely approximates the initial input values (1/1 mol/mol).

A comparison of the spectra of derivatives with the spectrum of unmodified polyamine reveals the emergence of intense bands at 1640–1650 cm^−1^, corresponding to the amide I (ν(C=O)) band, and bands around 1540 cm^−1^, characteristic of amide II (δ(N-H) and ν(C-N)). These bands are absent in the spectrum of the original polyamine, clearly indicating amide bond formation. The alterations in the configuration and amplitude of the intricate peak within the spectral range of 3000–2800 cm^−1^ serve as an indication of the presence of a fatty acid moiety in the analyzed substance. An increase in the intensity of peaks corresponding to O-H stretching absorption bands in the range of 3300–3500 cm^−1^ in the spectra of polyamine derivatives compared with unmodified polyamines may be due to a decrease in the hydration degree of and the formation of micelle-like structures due to the presence of fatty residues.

### 2.6. Influence of Polyamine and Its Derivatives on L-ASNase Internalization into Eukariotic Cell and Cytotoxicity

This study investigated targeted L-asparaginase delivery via the polyamine transport system (PTS), a pathway known to be highly expressed in rapidly dividing cancer cells, including certain lymphoma cell lines. The selection of K562 (human chronic myelogenous leukemia) and L5178Y (murine lymphoma) cells, both exhibiting significant PTS expression, allowed for a direct assessment of our approach’s efficacy in relevant target cells [32]. To evaluate selectivity and potential off-target effects, we included HEK293 cells, a commonly used model of normal cells, for comparison. A549 lung carcinoma cells, which express PTS at lower levels than the lymphoma cells, served as an additional control to further assess the target specificity of our delivery system. This comparative analysis using cell lines with varying PTS expression levels is crucial for demonstrating the targeted nature of our L-ASNase conjugate delivery.

#### 2.6.1. Fine Features of L-ASNase Interactions with Cancer Cells Studied Using FTIR Spectroscopy

**Figure 6:**
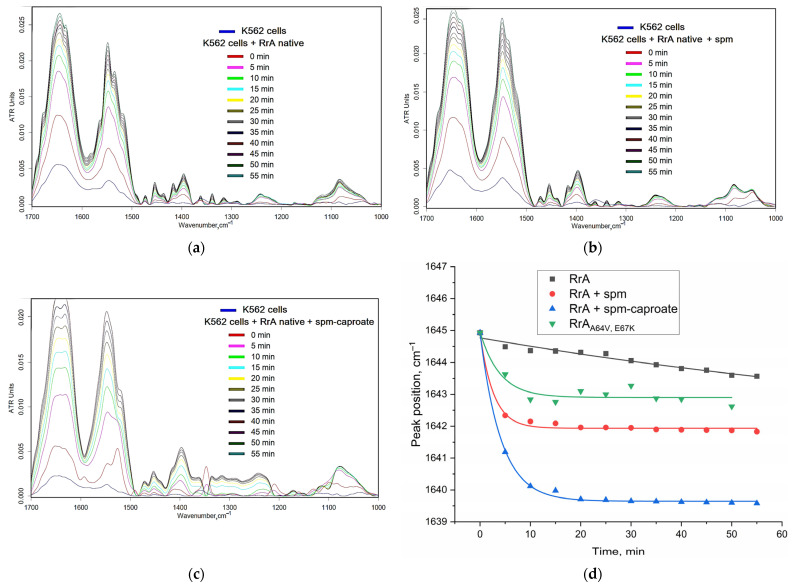
FTIR spectra of K562 cell suspension (1 × 10^7^ cell/mL, V = 30 uL) during real-time incubation with (**a**) RrA native 0.1 mg/mL, (**b**) RrA native 0.1 mg/mL + spm 1 mg/mL, and (**c**) RrA native 0.1 mg/mL + spm-caproate 1 mg/mL. (**d**) The dependence of the maximum position of the amide I peak in the spectra of K562 cells under the action of these formulations on time. PBS (0.01 M pH = 7.4). T = 37 °C.

To clarify the effect of polyamines and their derivatives on PTS as a way to regulate the interaction of the medically important enzyme L-ASNase with leukemic cells, we applied FTIR spectroscopy, which is a powerful tool for studying molecular composition and structural changes in biological systems, including cells (Figure 6). When examining the interaction between L-ASNase and K562 cells, FTIR spectra reveal the extent of this interaction and its impact on cellular components. The amide I band (1600–1700 cm^−1^) is sensitive to oscillations in the C=O bond in cell-membrane peptides, indicating protein secondary structure changes upon binding with ligands. Variations in its position, intensity, or width reflect protein conformational changes, interactions with cell-membrane lipids, or changes in cellular protein composition.

Figure 6d shows the relationship between the amide I peak position in K562 cell FTIR spectra and incubation time with different forms of asparaginase in the presence of polyamines or their derivatives. The interaction of native RrA with K562 cells is hardly discernible through a shift in the position of the peak (1 cm^−1^/1 h), as there are no specific ligands bound. The presence of spermine, a polyamine, modulates the effect of native RrA on the amide I region of the spectrum, causing more pronounced shift in its position (initial slope 3 cm^−1^/5 min). This implies that spermine enhances L-ASNase interaction with cell proteins and cell membrane via PTS. The addition of spm-caproate, a derivative of spermine, has a more pronounced effect on the position of the amide I peak (initial slope 4 cm^−1^/5 min) than spermine alone. Apparently, this is due to the presence of a hydrophobic tail, which disrupts the integrity of the membrane and enhances the interaction between the enzyme and the cell.

Interestingly, the mutant RrA (A64V, E67K), which we previously examined in terms of introducing positive charges at the interface between subunits due to the presence of lysine [21], exhibits a significantly distinct dynamic behavior compared to its native form. This disparity can be attributed to the presence of lysine. The inclusion of lysine not only imparts a positive charge but also brings about structural alterations that enhance the interaction of the enzyme with the cell membrane. This, in turn, results in an increase in cytotoxic activity compared to native RrA [21].

#### 2.6.2. Quantification of L-ASNase Interactions with Cancer Cells Studied Using Fluorimetry

The influence of competitive inhibitors (free polyamines) on enzyme permeability was investigated using the fluorometric detection of eosin-labeled enzyme or polyamine (Figure S2). Fluorimetry quantified the interaction of native enzymes, their conjugates, and polyamines with K562 leukemia and A549 adhesion cancer cells, both with and without added polyamines or their derivatives. This approach aimed to elucidate the functional consequences of polyamine conjugation to the enzyme, the role of non-covalent polyamine additives, and the involvement of PTS. Table 2 presents data on the binding of native EcA, its polyamine conjugate EcA-spd, native EwA, and its conjugate EwA-spd, as well as the free polyamines spermine (spm) and polyethyleneimine (PEI), to K562 and A549 cancer cells.

In both cell lines, polyamine conjugation consistently increases the binding of native L-ASNase (up to 30–40%). For example, EcA-spd exhibits higher binding than native EcA in both K562 and A549 cells. This suggests that the polyamine moiety facilitates interaction with the cell surface, potentially through the PTS. A similar trend is observed with EwA and EwA-spd.

The degree of polyamine interaction with various cell types, for example, K562 and A549, might be indicative of discrepancies in the levels of expression or activity of PTS across these cell lines. Specifically, unmodified spm (the original ligand for PTS) exhibits a significantly higher binding affinity for A549 cells, with a maximum of 46%, compared to K562 cells, where the maximum binding is only 21%. Conversely, PEI demonstrates a rather non-specific binding pattern, likely due to its multipoint interaction with the cell surface.

The addition of polyamines and their derivatives significantly modulates the binding of L-ASNase and its conjugates, highlighting the complexity of PTS interactions. In many cases, the addition of polyamines or their derivatives appears to compete with the enzyme conjugates for binding, leading to reduced binding with the cells. This competitive inhibition is evident with the addition of spm-caproate and spm-lipoate, which generally decrease the binding of enzymes and conjugates in both cell lines. In some other cases, added polyamines enhance binding, possibly by upregulating or stimulating PTS activity. For example, the addition of spd to A549 cells notably increases the binding of EwA. 

#### 2.6.3. The Effect of Polyamines and Their Derivatives on the Cytotoxicity of L-ASNase Towards Cancer Cells

The ability to modulate (compete or potentially synergistically enhance) the cytotoxicity of L-ASNase through polyamine manipulation is crucial for improving treatment outcomes in leukemia. Table 3 shows the survival rates of K562 leukemia and A549 adhesion cancer cells upon treatment with native EcA, spermine-conjugated EcA (EcA-spm), and the influence of added polyamines and their derivatives on cell proliferation, as well as cell viability data for both cell lines after treatment with various polyamine derivatives.

**Efficacy of Individual Components**

Initially, we established the intrinsic activity of each agent. Polyamine derivatives alone generally exerted a mild to moderate cytostatic effect on both cell lines. For instance, X-caproate reduced A549 viability to 60–70%, while X-lipoate was notably potent with Spd for A549, achieving 23% viability. Native EcA consistently demonstrated a cytostatic effect, resulting in 67% viability for A549 cells and 58% for K562 cells. The EcA-spm conjugate alone proved slightly more potent, reducing A549 viability to 58% and K562 to 50%, suggesting that the conjugation enhanced the enzyme’s intrinsic efficacy or cellular uptake.

**Interaction Patterns of Native EcA in Combination with Polyamines**

When native EcA was combined with polyamine derivatives, distinct trends in interaction emerged. In the most cases, in the presence of polyamines the increased cytostatic activity is observed probably due to the non-specific influence of the polyamines on the membrane permeability. For A549 cells, the combinations of EcA with Spd (45% viability) and PEI (31% viability) showed synergy effect compared to  58% A549 viability for EcA. Caproate derivatives mainly showed additive effect for EcA + PEI-caproate (31%)., but EcA + PEI-lipoate yielded a remarkable 16% viability, making it a highly effective enhancer for A549 cells. However, a consistent antagonistic trend was observed with Spm and its derivatives (the main specific ligand to PTS): EcA + Spm resulted in 97% observed viability. Similar trends were seen in K562 cells: synergy with Spd (39%) and PEI (36%), but reduced efficacy with Spm (66%).

**Interaction Patterns of EcA-spm Conjugate in Combination with Polyamines**

For polyamine-conjugated enzymes the presence of competitive ligands to PTS receptors mainly decreased the uptake and cytostatic activity. The screening for competing inhibitors of PTS-mediated endocytosis revealed spermine and caproate/lipoate derivatives as the most potent inhibitors or antagonists, significantly reducing the cytostatic efficacy of polyamine-conjugated ASNases- fatty acid derivatives of polyamines produced the strongest suppression. 

For K562 cells, the overall trend for EcA-spm combinations was an inhibitive effect. For EcA-spm + Spd-caproate or PEI-lipoate (up to 101% cells viability observed relative to control), highlighting the concurrent effect of the polyamine to the PTS receptors.

Simultaneously with this suppression, in some cases, competitive ligands to PTS significantly promoted the uptake of the native non-conjugated enzymes, “equalizing” the cellular access and cytostatic activity for native vs conjugated ASNase. The results obtained emphasize the complex, cell-type-dependent and inhibitor-specific nature of these interactions, which highlights the profound involvement of PTS in L-ASNase internalization and cytotoxic activity. 

For A549 cells different picture is observed: combinations with unmodified polyamines mainly show additive effect: for EcA-spm with PEI or PEI-caproate, PEI-propionate achieving 19-25% observed viability with PEI. This consistently positioned PEI and its derivatives as an effective non specific enhancer for enzyme conjugates (EcA-spm) against A549 cells with non pronounced overexpressed PTS receptors compared to K562 leukoses cells. However, lipoate derivatives with EcA-spm were indifferent, despite some numerically effective combinations like EcA-spm + PEI-lipoate (26%). 

**Polyamine Type Specificity**

**Spm**, both in its unmodified form and sometimes its derivatives, mainly acted as an concurrent inhibitor of asparaginase internalization and cytotoxic, leading to higher observed cell viabilities compared to other polyamines. In contrast, **Spd** and especially **PEI** consistently emerged as enhancers due to its non-specific effect on the membrane permeability. Combinations involving Spd and PEI, particularly with native EcA, mainly resulted in synergistic or additive effects, leading to the lowest observed cell viabilities.

**Cell-Line Specificity**

Sspecific quantitative differences and interaction types were observed between A549 and K562 cells. For example, the overall classification for EcA-spm combinations show concurrent effect against K562, but additive or indifferent effect is observed for A549. This finding corroborates the hypothesis that the interaction between K562 cells and concurrent agents is contingent on the presence of PTS, whereas the same cannot be said for A549 cells.

**Figure 7:**
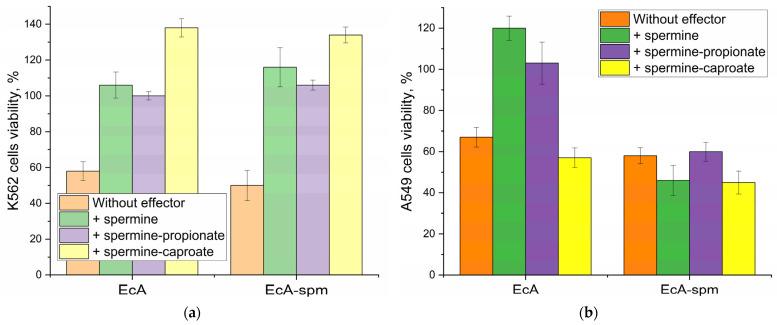
A schematic representation of the cytostatic potency of EcA and its derivative EcA-spm when exposed to various effectors on the (**a**) K562 cell line (PTS-positive) and (**b**) A549 cell line (PTS negative) (conditions indicated in Table 3). The impact of L-asparaginase is provided, taking into account the contribution of oligoamines.

Figure 7 illustrates the distinct modulatory effects of spermine and its derivatives on L-asparaginase cytostatic activity, significantly influenced by cellular PTS status. In K562 (PTS-positive) cells, spermine and its propionate/caproate derivatives consistently acted as potent antagonists, drastically increasing viability for both native EcA and the EcA-spm conjugate, suggesting interference for PTS. Conversely, in A549 cells, free spermine remained inhibitory with native EcA, the caproate derivative transformed into an effective adjuvant for both EcA and EcA-spm, indicating that the non pronounced functional PTS (in the case of A549) alters interaction mechanisms and can lead to synergistic outcomes depending on the specific polyamine modification and enzyme form. This consistent enhancing effect highlights the potential of spermidine and its propionate as beneficial co-therapeutics. Therefore one can reveal the most compelling formulations identified for A549 cells: **EcA + PEI-lipoate** (16% viability), EcA-spm with PEI or PEI-caproate (both 19% viability). These findings highlight the potential of combination therapies and the importance of derivative-specific and cell-line-specific responses in developing effective cancer treatments.

#### 2.6.4. Correlation of Binding Data with Cytotoxicity Data of L-ASNases

**Figure 8:**
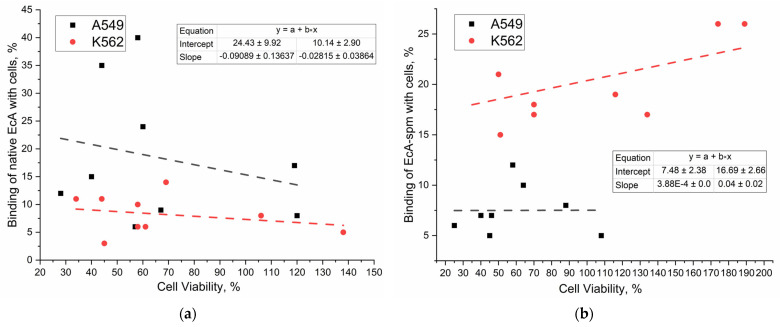
Correlation graphs of the binding data from Table 2 with the cell viability data from Table 3 for (**a**) for EcA formulations; (**b**) for EcA-spm formulations. The presented samples are from Table 3.

To investigate the relationship between asparaginase uptake and therapeutic efficacy, we correlated binding data (Table 2) with cell viability data (Table 3). This analysis, visualized in Figure 8, reveals the impact of cellular uptake on the observed cytostatic activity. Furthermore, the inclusion of permeability inhibitors demonstrates a general reduction in both cellular uptake and cytostatic activity (Table 3), underscoring the importance of efficient cellular entry for effective therapeutic action.

We investigated the modulation of enzyme–cell binding through two key approaches: (1) the covalent conjugation of the enzyme with polyamines to alter its binding affinity and (2) competitive effector using free polyamines as ligands for the PTS to modulate binding indirectly. Figure 8a,b depicts the correlation between the cellular binding of different asparaginase formulations and their resulting cytostatic effects, normalized to isolate the enzyme’s contribution. A comparison of the PTS-positive K562 cells and the A549 cells, exhibiting lower PTS receptor expression, reveals crucial differences. In K562 cells, a positive correlation is observed between binding and cell viability across all formulations, indicating that increased binding provides the increased viability. This suggests that factors beyond simple binding, potentially related to intracellular trafficking or enzyme activity, significantly influence the cytostatic effect in these cells. In contrast, A549 cells display a more complex relationship which include the non-specific effect of PEI on the membrane permeability. This highlights the importance of PTS expression in mediating the cytotoxic effects of asparaginase. The observation of a negative correlation for native EcA in A549 cells further emphasizes this point, suggesting that in cells with lower PTS expression, increased binding may be less effective in achieving a cytostatic outcome. Notably, the EcA-spm conjugate exhibits a strikingly different behavior compared to native EcA in both cell lines. The near-zero correlation in K562 cells for EcA-spm suggests that the spermine modification alters the relationship between binding and cytotoxicity, potentially by affecting intracellular localization or interactions with other cellular components. The contrasting responses between the two cell lines clearly demonstrate the influence of PTS expression on the efficacy of asparaginase formulations and the impact of the spermine moiety as an effector.

#### 2.6.5. Mechanisms of Internalization of Native and Polyamine-Conjugated Asparaginases into Cancer Cells

Understanding the internalization mechanisms of polyamine-conjugated L-ASNase, particularly their interaction with PTS receptors, is crucial for comprehending L-ASNase function, intracellular distribution, and avenues for enhancing its therapeutic efficacy. Significant differences are observed between the uptake pathways of native and polyamine-conjugated enzymes. Recently, our colleagues conducted an in-depth study. Recent studies of endocytosis on caveolae, clathrin, and micropinocytosis-mediated systems to analyze the uptake of asparaginase preparations have shown that native EcA and EwA exhibit low levels of cellular uptake, suggesting the antiproliferative effect is mainly due to extracellular asparagine depletion. In contrast, cytoplasmic enzyme RrA displays notable internalization via clathrin-dependent endocytosis: the inhibition of clathrin system reduces RrA cellular interaction, while no effect was found with the inhibition of caveolin receptors [60].

**Figure 9:**
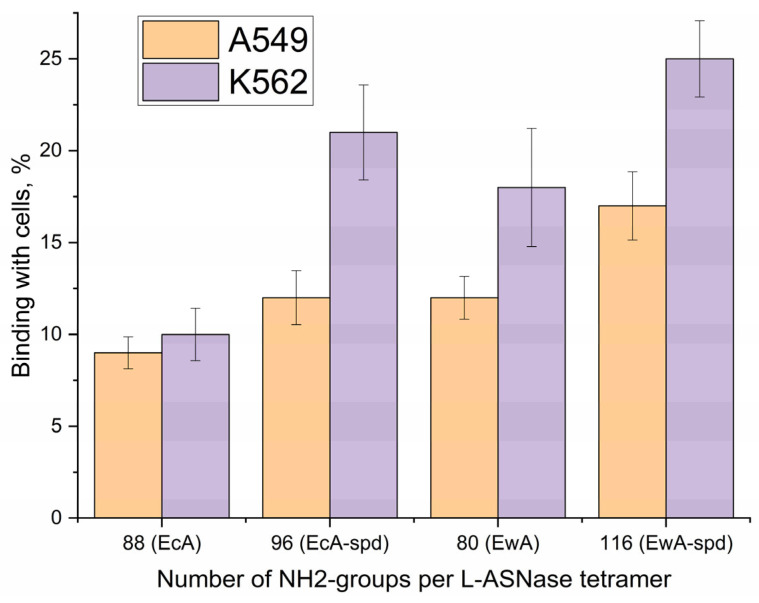
Correlation between the number of NH2-groups per L-ASNase tetramer with the percentage of L-ASNase bound with cells.

Polyamine conjugation alters the internalization mechanism of L-ASNase. Polyamines can interact with the heparan sulfate (HSPGs) residues in glypican 1 (GPC1) coordinately transport spermine and enter into the cell. Alternatively, polyamine transport is mediated by endocytosis, where polyamines bind to polyamine-binding proteins. For instance, recent studies have shown that ATP13A2 promotes the uptake of polyamines by cells through endocytosis and transports them to the cytoplasm, highlighting the role of endolysosomes in the uptake of polyamines into cells [39]. Our findings indicate that polyamine-conjugated enzymes enter cells by PTS-targeting endocytosis. This conclusion is supported by data presented in Figure 9. A good correlation was observed between the number of polyamine residues attached to the enzyme and cellular binding for both K562 and A549 cells. Conjugation consistently enhances cellular internalization across different L-ASNase types, with the effect proportional to the number of added spermine moieties (and thus amino groups). Moreover, competitive inhibitors of PTS (spm-lipoate) effectively block this enhanced uptake (Table 3). This directly implies a specific PTS-mediated endocytosis pathway. The differential effects of PEI, spermine, and spermidine on asparaginase internalization further underscore the specificity of this interaction, demonstrating rather a specific engagement with PTS receptors.

The contrasting uptake routes highlight that polyamine conjugation not only enhances cellular internalization but also shifts the dominant endocytic pathway, potentially influencing the intracellular fate and therapeutic efficacy of the enzyme.

### 2.7. Visualization of the Polyamine Transport System’s Role in L-ASNase Delivery Using CLSM

The L5178Y cell line, a well-established model for lymphoma research and asparaginase studies, was chosen to ensure the relevance of our preclinical findings to potential clinical applications. Its consistent use in prior research provides a robust basis for the comparison and validation of our results within the broader scientific literature.

**Figure 10:**
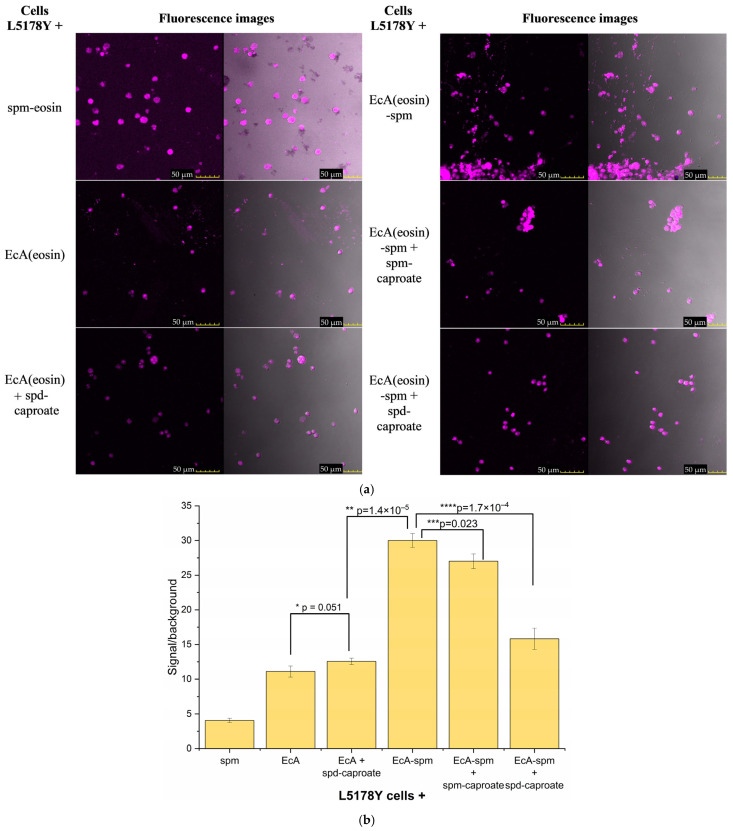
(**a**) CLSM images of L5178Y lymphoma cells treated with L-ASNase or polyamine samples labeled with eosin. In the case of L-ASNase conjugates, the protein was labeled with eosin. λexci, max = 515 nm, λemi = 530–570 nm. The scale bar is 50 µm. (**b**) Signal/background fluorescence ratios for these formulations.

Figure 10 presents data on the cellular uptake and localization of L-asparaginase (L-ASNase) and polyamine conjugates in L5178Y lymphoma cells using confocal laser scanning microscopy (CLSM). Figure 10 comprises (a) CLSM images and (b) quantitative fluorescence signal/background ratios.

The signal/background (S/B) ratio for eosin-labeled EcA (S/B = 11) suggests moderate internalization of the native enzyme. This confirms cellular uptake, but the level is not as high as the conjugated form, potentially suggesting limitations in natural uptake mechanisms. EcA-spm exhibits a significantly higher signal/background ratio (S/B = 30) compared to native EcA. This strongly suggests that polyamine conjugation substantially enhances the cellular uptake of L-ASNase, likely through interactions with polyamine transporters. This observation supports the rationale for developing polyamine-conjugated L-ASNase for improved therapeutic efficacy.

The addition of polyamine derivatives influences the uptake of both native and conjugated L-ASNase. The relatively low signal/background ratio for spm-eosin (S/B = 4) indicates the limited uptake of free spermine under these experimental conditions. Adding spd-caproate to native EcA slightly increases the signal/background ratio (S/B = 12), which might indicate some degree of uptake facilitation, though the effect is minimal. However, adding spm-caproate or spd-caproate to EcA-spm decreases the signal/background ratio (S/B = 26 and 16, respectively) compared to EcA-spm alone. This suggests that these oligoamine derivatives, compete with the Enzyme conjugate for uptake and interfere with the internalization process.

Our findings indicate that EcA-spm is internalized via PTS, while unconjugated EcA cellular uptake is enhanced by PTS ligands, suggesting membrane permeability modulation. Fatty acid derivatives of Spd and Spm significantly amplify competitive inhibition for EcA-spm’s PTS binding, thereby reducing its effect. Conversely, these same derivatives enhance the cellular uptake of native EcA. Both free polyamines and polyamine-conjugated proteins are actively internalized by cells. These complex interactions highlight the potential for improved leukemia therapies through strategic combinations of L-ASNase with specific polyamine transport inhibitors and optimized polyamine-conjugated enzymes.

### 2.8. Selectivity of L-ASNases Against Cancerous Cells vs. Normal

A comparative analysis of the specificity of action exhibited by native asparaginases and asparaginase conjugated with polyamines in relation to cancerous cells, in contrast to their effect on healthy cells. The selection of K562 (leukemia) and L5178Y (lymphoma) cells, both exhibiting significant PTS expression, allowed for a direct assessment of our approach’s efficacy in relevant target cells. To evaluate selectivity and potential off-target effects, we included HEK293 cells, a commonly used model of normal cells, for comparison.

**Figure 11:**
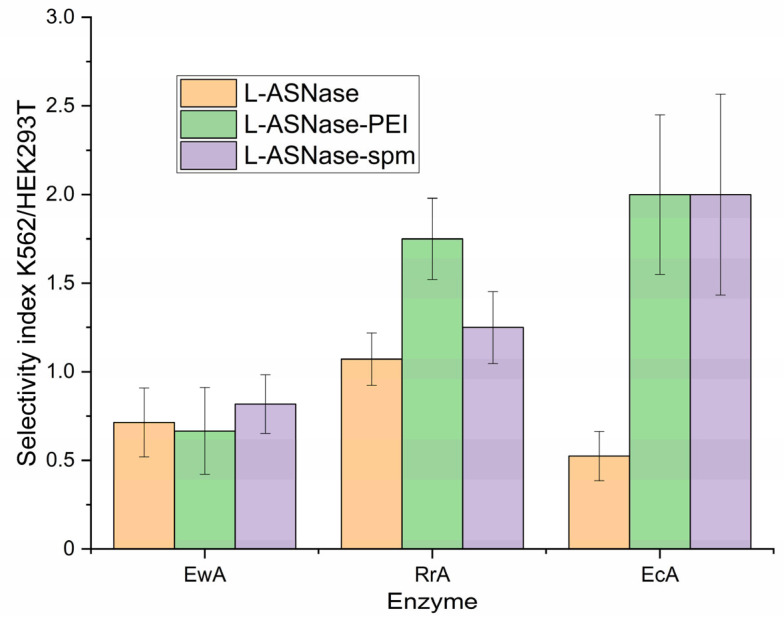
Selectivity indices of the action of native and polyamine-conjugated L-ASNases on cancer cells vs. normal (according to survival by MTT analysis). RPMI-1640. 5% CO_2_. T = 37 °C. For EwA, there are statically insignificant differences between the native enzyme and conjugates (*p*-value > 0.05), for RrA, *p*-value < 0.05 for conjugate with PEI, and for EcA, *p*-value < 0.001 for all conjugates.

The data presented in Figure 11 highlights the selective action of polyamine-conjugated asparaginases compared to their native counterparts against various cancer cell lines, but here is a specific example of K562, in relation to normal HEK293T cells. This selectivity is quantified through the selectivity index (K562/HEK293T), showcasing the differential efficacy of the enzymes.

The results indicate that while the native L-ASNase exhibits moderate selectivity, the polyamine-conjugated forms, particularly L-ASNase-PEI and L-ASNase-spm, demonstrate significantly enhanced selectivity indices. For instance, EcA and RrA variants of L-ASNase-PEI and L-ASNase-spm exhibit selectivity indices of 2, indicating their superior capacity to preferentially target and affect cancer cells over normal HEK293T cells.

This enhanced selectivity is a promising characteristic of polyamine-conjugated asparaginases, suggesting that they can more effectively exploit the unique metabolic environments of cancer cells while minimizing toxicity to healthy cells. The ability of these conjugates to demonstrate a favorable selectivity profile may improve therapeutic outcomes in cancer treatment, making polyamine-conjugated asparaginases a valuable area of research for developing targeted therapies.

## 3. Materials and Methods

### 3.1. Chemicals

L-asparaginase fluorescent substrate L-aspartic acid β-(7-amido-4-methylcoumarin) (Asp-AMC) and the product 7-amino-4-methylcoumarin (AMC) were purchased from Sigma-Aldrich (St. Louis, MO, USA). Polyamines for enzyme modification, including polyethyleneimine 2 kDa (PEI2), spermine (spm), spermidine (spd), and putrescine (put) were also obtained from Sigma-Aldrich (St. Louis, USA). Heparin with a molecular weight range of 12–14 kDa was used for medical purposes and was intended for intravenous administration. Activated derivatives of carboxylic acids were obtained from Reachim production (Moscow, Russia).

Other compounds utilized in the study included 1-ethyl-3-(3-dimethylaminopropyl) carbodiimide (EDC), N-hydroxysuccinimide (NHS), salts, acids, and buffer components, which were procured from Reachim production (Moscow, Russia).

### 3.2. L-Asparaginases

Escherichia coli L-ASNase (EcA) was acquired from Veropharm^®^ (Moscow, Russia). The L-ASNase from Rhodospirillum rubrum (RrA) was obtained as previously detailed [21,78]. Recombinant L-ASNase from Erwinia carotovora (EwA) was prepared as described earlier [79]. The initial activity of L-ASNases was measured using standard protocol circular dichroism spectroscopy on a J-815 CD spectrometer (Jasco, Tokyo, Japan) [80].

### 3.3. Synthesis of Polyamine-Conjugated L-ASNase

L-ASNase conjugates were synthesized using carbodiimide chemistry. L-ASNase (1 mg/mL) in 0.1 M phosphate buffer (pH 6.0) was activated with EDC and NHS (dissolved in acetonitrile at a concentration of 30 mg/mL) at a weight ratio of 0.4 and 0.2, respectively, relative to the enzyme. After a 30 min incubation at 35 °C, a solution of polyamine in the same buffer was added dropwise to the activated L-ASNase until a specific (as depicted in Table 1) enzyme-to-polymer molar ratio was achieved. The reaction mixture was then incubated for an additional 2 h at 35 °C. The resulting conjugates were purified by dialysis (12–14 kDa cutoff) against phosphate-buffered saline (PBS) at 4 °C for 24 h (two 12 h exchanges), lyophilized, and stored for further use. The composition and stoichiometry of the conjugates were determined using circular dichroism (CD) and Fourier-transform infrared (FTIR) spectroscopy (Figure 2).

We have determined the extent of enzyme modification by titrating amino acids using the trinitrobenzene sulfonic acid (TNBS) reagent. A brief protocol for quantifying the number of amino groups involves the following steps: To 200 µL of solutions containing enzymes, their conjugates, or oligoamines at a concentration ranging from 1 to 15 µg/mL in borate buffer (pH 9.2, 20 mM), we added 2 µL of a 1 M solution of TNBS. Kinetic absorption curves were then recorded at a wavelength of 420 nm. The quantity of amino groups was calculated relative to unmodified enzymes and oligoamines, with spermine serving as a standard. Spermine is known to undergo titration with two primary amino groups, making it an appropriate reference point for comparison.

### 3.4. Synthesis of Polyamine Derivatives

Polyamine derivatives were synthesized as shown in Figure 5a. To do this, an activated acid solution (20 mg/mL) in DMSO was slowly added dropwise to the polyamine solution (1 mg/mL) in PBS until an equimolar ratio was reached. Purification was performed using anti-water dialysis (cut-off 300–500 Da), then lyophilized, and stored for further use. Degrees of modification by fatty acids 1.1 mol/mol.

### 3.5. FTIR Characterization of Polyamine-Conjugated L-ASNases and Polyamine Derivatives

FTIR spectra of native enzymes, polyamine-conjugated L-ASNases and polyamine derivatives were acquired using either a MICRAN-3 FTIR microscope (Simex, Novosibirsk, Russia) or a Bruker Tensor 27 spectrometer (Bruker Optics, Ettlingen, Germany) equipped with a liquid-nitrogen-cooled MCT detector.

### 3.6. Determination of L-ASNase Catalytic Activity

L-ASNase activity was assessed by incubating the enzyme formulation (0.1–100 µg/mL final concentration) with a 1 mM solution of Asp-AMC in 0.01 M PBS (pH 7.4). After mixing, the kinetic curves were monitored fluorometrically (excitation 360 nm, emission 460 nm). Enzyme activity (U/mg) was calculated from the initial linear portion of the kinetic curves (0–10 min), using a standard EcA preparation as a reference.

### 3.7. Cell Studies

#### 3.7.1. Eukaryotic Cell Cultures

L5178Y lymphoma cells, K562 leukemia cells, A549 lung carcinoma cells and HEK293T cells were obtained from the Cell Collection of the Lomonosov MSU in Moscow, Russia. The cells were cultured in RPMI-1640 medium (Gibco, Thermo Fisher Scientific, Waltham, MA, USA), supplemented with 5% fetal bovine serum (Capricorn Scientific, Marburg, Germany) and 1% sodium pyruvate (Paneco, Moscow, Russia). The cells were maintained in a humidified incubator at 37 °C under conditions of 5% CO_2_.

#### 3.7.2. Fluorescence Determination of the Binding of L-Asparaginase Formulations to Cells in the Presence of Modulators

The process of sample preparation commenced with the covalent attachment of eosin isothiocyanate (1 μg/mL) to L-ASNase (1 mg/mL), resulting in the formation of a conjugate known as L-ASNase-eosin (Figure S2). Subsequently, EwA-eosin underwent modification to generate conjugates with polyamines utilizing the carbodiimide method.

A fixed volume of each L-asparaginase solution was subsequently added to a suspension of K562 or A549 cells, with a concentration of 100,000 cells per 100 μL. These mixtures were incubated at 37 °C to allow for proper interaction. Finally, the fluorescence intensity was measured using a SpectraMax M5 instrument (Sunnyvale, PA, USA) on a 96-well Costar plate with a black and transparent bottom.

### 3.8. FTIR Spectroscopy for Studying Enzyme-Cell Interactions

Cell suspensions containing 4–7 × 10^5^ cells per milliliter were centrifuged at 4000× *g* for 2 × 5 min using an Eppendorf centrifuge model 5415C. The cells were then washed twice with sterile phosphate-buffered saline (PBS) at a pH of 7.4. The cell pellet was resuspended in PBS to a concentration of 1 × 10^7^ cells per milliliter. A 20 μL aliquot of the cell suspension was placed on the spectrometer chamber, followed by the addition of 10 μL of an enzyme solution with a concentration of 1–5 mg per milliliter. The samples were incubated at 37 °C, and Fourier-transform infrared (FTIR) spectra were recorded at 5 min intervals over a period of one hour on a Bruker Tensor 27 spectrometer (Bruker Optics, Ettlingen, Germany) equipped with a liquid-nitrogen-cooled MCT detector. The settling of cells without the addition of the drug is considered as a background control.

FTIR mapping of integral absorbance of polyamine labeling PTS receptors on L5178Y cells was performed using MICRAN-3 FTIR microscope (Simex, Novosibirsk, Russia). Scanning step was 2 µm. Eosin was necessary for the visual identification of cells.

### 3.9. Determination of Cell-Associated Enzyme Concentration

The concentration of native enzymes and its conjugates (enzymes bound to cells and enzymes adsorbed to the cell surface) was quantified fluorometrically using eosin covalently linked to L-ASNase. Enzyme-eosin conjugates (0.5 mg/mL) were incubated for three hours at 37 °C with L5178Y lymphoma cells, K562 leukemia cells, and A549 lung carcinoma cells at a concentration of 2 × 10^6^ cells/mL. Following incubation, the cells were separated by centrifugation at 5000× *g* for five minutes. The fluorescence intensity of the supernatant and the cell suspension was measured using excitation and emission wavelengths of 515 nm and 550 nm, respectively. From these measurements, the percentage of cell-associated enzyme was calculated.

### 3.10. Cytotoxicity Assay

Cytotoxicity was assessed using a modified MTT (3-(4,5-dimethylthiazol-2-yl)-2,5-diphenyltetrazolium bromide) assay. The procedure involved seeding cells at a predetermined density in 96-well microtiter plates. After 24 h of incubation, the cells were exposed to various concentrations of the test compounds. Following an additional 48 h incubation period, MTT solution was added to the wells, and the cells were incubated for another 12 h. The formazan crystals formed were subsequently dissolved in a dimethyl sulfoxide (DMSO) solubilization solution. The absorbance of the resulting solution was measured using a microplate reader at a wavelength of 570 nm. The absorbance values were directly proportional to the number of viable cells.

### 3.11. Investigation of the L-ASNase Interactions with Cancer Cells Using Confocal Laser Scanning Microscopy

L5178Y lymphoma cells, K562 leukemia cells, A549 lung carcinoma cells, and HEK293T cells were incubated for 4 h with eosin-labeled enzyme or polyamine formulations (0.05 mg/mL). Eosin was visualized using 515 nm excitation and 530–570 nm emission. CLSM images were obtained using a FluoView FV1000 microscope (Olympus, Tokyo, Japan), equipped with a spectral scan unit and transmitted light detection. This CLSM system was integrated with the IX81 inverted microscope. The UPlanSApo 20×/0.75 objective provided an axial resolution of ≤2 μm at 580 nm, sufficient to clearly visualize fluorophores localized within cells rather than merely on the surface. Image acquisition was performed using FV10 ASW software (version 1.7, Olympus), and subsequent image processing was conducted using ImageJ software (version 1.53e, NIH).

### 3.12. Atomic Force Microscopy

Atomic force microscopy (NTEGRA II AFM microscope, NT-MDT Spectrum Instruments, Moscow, Russia) was used to visualize of L5178Y cells after treatment with PEI2 showing clustering of polyamine receptors.

## 4. Conclusions

This study elucidates the influence of polyamines, as competitive inhibitors, on the binding, permeability, and the cytostatic efficacy of both native L-asparaginase (EcA) and its polyamine-conjugated enzyme (EcA-spm) on tumor cells.

Our findings reveal a complex, dualistic role for polyamines. While native EcA, generally exhibits limited direct cellular internalization, its permeability into target cancer cells is notably enhanced in the presence of free polyamines. Conversely, poly-amine-conjugated EcA-spm relies significantly on the PTS for its cellular uptake and subsequent cytostatic action. Specific ligands free polyamines and the derivatives acting as competitors for PTS receptors, significantly inhibiting the binding and internalization of EcA-spm. This competitive interaction can lead to a phenomenon where the addition of free oligoamines effectively “equalizes” the cellular access properties of both native EcA and EcA-spm. When PTS is saturated or competitively blocked by high concentrations of polyamines, both forms of L-ASNase face hindered entry through PTS, thereby modulating their intracellular availability and efficacy.

To further amplify these competitive effects of polyamines, we synthesized fatty acid derivatives of oligoamines. These modifications were specifically engineered to enhance the inhibitory capacity of polyamines towards PTS receptors, primarily through increased lipophilicity facilitating enhanced membrane interaction and potentially higher intracellular accumulation, thereby providing a more robust modulation of L-ASNase and its conjugate’s cellular entry.

Our observations suggest that the presence of polyamine inhibitors consistently resulted in a decrease in the cytostatic efficacy of L-ASNase conjugates. In the case of EcA-spm, an increase in cellular survival (decreased cytotoxicity) was mainly observed in the presence of these polyamine inhibitors. Specifically, unmodified spermine significantly increased cellular survival when tested for EcA-spm by 22% (, from 50% to 72%), while having only a marginal effect on native EcA (+8%). Even more strikingly, Spd-caproate completely abrogated EcA-spm cytotoxicity on K562 cells, boosting sur-vival from 50% to 101%, whereas it had a slightly synergistic effect to native EcA. This differential response confirms that polyamines and their derivatives significantly diminish the acquired PTS-dependent cytotoxicity of EcA-spm by competing for uptake pathways. Conversely, specific polyamine derivatives, particularly lipoate and PEI conjugates, proved highly effective at enhancing L-ASNase activity. Notably, the co-administration of EcA or EcA-spm with Spd-lipoate and PEI-caproate achieved the lowest cell survival rates (15-20% viability against A549), representing the most potent formulations and demonstrating strong synergistic effects for the conjugated enzyme. This potentiation likely stems from a multi-faceted mechanism: PEI’s polycationic na-ture can induce membrane permeabilization and facilitate endosomal escape, thereby increasing intracellular enzyme delivery. However, PEI-caproate and spd-lipoate have intrinsic cytotoxicity. Taking this into account, leads to conclusion that their presence decreases the contribution of conjugated ASNase in “gross” cytotoxicity. 

In essence, our research provides compelling direct evidence that covalent poly-amine modification significantly enhances L-ASNase cellular uptake and cytotoxicity, primarily through a PTS-dependent mechanism. Furthermore, co-administration of L-ASNase with select PTS ligands, such as PEI and its fatty acid derivatives, synergisti-cally boosts these anti-cancer effects by modulating membrane permeability and intra-cellular delivery pathways, potentially leading to a more pronounced therapeutic out-come. This deepened understanding is paramount for the rational design of next-generation L-ASNase therapies that selectively harness and precisely modulate the often-overexpressed PTS in cancer cells, ultimately overcoming current therapeutic limitations and paving the way for more selective and profoundly effective anti-cancer treatments.

## Figures and Tables

**Table 1:** Physico-chemical properties of L-ASNases. L-ASNase kinetics parameters were determined by CD spectroscopy using Asn as a substrate (*n* = 3, data presented as a mean ± SD), PBS (0.01 M, pH 7.4)—data from [22]. T = 37 °C. [S]_0_ = 10 mM. The characteristics of conjugates of L-ASNases with oligoamines—molar ratios of polyamine: protein tetramer.

Enzyme	Type	Quaternary Structure	M_W_ (Monomer), kDa	pH-Optimum	Specific Activity of Native Enzyme, U/mg	Molar Ratios of Polyamine: Protein Tetramer Used in Synthesis and Determined Using TNBS Titration in Parentheses			
						Spermine (spm)	Spermidine (spd)	Putrescine (put)	PEI2
*RrA*	Ia	Tetramer/dimer	18	9.0–9.3	60 ± 6	2; 5; 10; 30 (2; 5; 9; 25) *	2; 5; 10; 30 (2; 4; 9; 23) *	2; 5; 10; 30 (2; 5; 8; 22) *	0.2; 0.5; 2; 5 (0.2; 0.5; 1.8; 4) *
*EwA*	II	Tetramer	34.2	8–9	370 ± 15	5; 10; 20; 50 (4; 8; 17; 42) *	5; 10; 20; 50 (4; 9; 16; 36) *	5; 10; 20; 50 (4; 8; 14; 35) *	0.5; 1; 3; 5 (0.4; 0.8; 2.6; 4.5) *
*EcA*	II	Tetramer	36.9	8–8.5	330 ± 20	5; 10; 20; 50 (3; 6; 15; 38) *	5; 10; 20; 50 (4; 8; 17; 39) *	5; 10; 20; 50 (4; 7; 18; 40) *	0.5; 1; 3; 5 (0.5; 0.9; 2.5; 4.2) *

* Determined using TNBS titration of amino groups.

**Table 2:** The proportion of bound native enzymes, their conjugates, or polyamines with cancer leukemia cells K562 and cancer adhesion cells A549 with the addition of 0.1 mg/mL polyamine or its derivatives in comparison with non-treated cells for 2 h of incubation. Enzymes or polyamines were eosin-labeled for fluorometric detection (λexci = 515 nm, λemi = 550 nm). RPMI-1640. 5% CO_2_. T = 37 °C. The errors (SD) obtained as a result of three independent measurements did not exceed 15%.

Enzyme or Polyamine		% Binding with A549 Cells *						% Binding with K562 Cells *					
		*EcA*	*EcA-spd*	*EwA*	*EwA-spd*	*spm*	*PEI*	*EcA*	*EcA-spd*	*EwA*	*EwA-spd*	*spm*	*PEI*
Typical Effect on the Enzyme (Polyamine) Binding with Cells **		+	−	**0**	+	**0**+	**0**	**0**−	**0**	**0**−	**0**−	+	**0**
**Effector**	**Without effector**	**9**	**12**	**12**	**17**	**11**	**15**	**10**	**21**	**18**	**25**	**15**	**16**
	Spm	8	7	10	25	46	39	8	19	17	21	14	15
	Spm-caproate	6	5	7	21	6	12	5	17	8	13	11	13
	Spm-lipoate	24	5	7	23	8	17	6	18	9	17	13	20
	Spd	40	7	17	37	13	38	3	15	14	30	31	35
	Spd-laurate	35	10	11	20	13	12	11	24	18	24	22	16
	Spd-caproate	17	8	17	24	20	17	11	26	14	13	22	17
	PEI4	15	6	11	21	16	17	6	17	11	20	20	16
	PEI4-lipoate	12	27	23	14	11	10	14	26	20	21	37	16

* The error of determination did not exceed 20%. ** The symbol «+» denotes an increase in intensity, «−» represents a decrease, «0» indicates no change, «0+» denotes a weak positive change, and «0−» denotes a weak negative change.

**Table 3:** Survival rate of lung cancer adhesion cells A549 and leukemia cells K562 under the action of native EcA 5 U/mL and polyamine-conjugated EcA-spm 5 U/mL with addition of 0.1 mg/mL polyamine derivatives in comparison with non-treated cells. Survival rate of cancer leukemia cells K562 and cancer adhesion cells A549 under the action of polyamine derivatives (0.1 mg/mL) in comparison with non-treated cells. In parenthesis for combined formulations, the impact of L-asparaginase is provided, with the exclusion of the contribution from oligoamines or their derivatives. The cumulative effect of these two constituents is then indicated in parentheses. T = 37 °C. The errors (SD) obtained as a result of three independent measurements did not exceed 15%.

Cell Viability *, %	The Effect on Cell Growth	The Type of the Interaction with the Enzymes	A549			K562		
			*X = Spm*	*X = Spd*	*X = PEI*	*X = Spm*	*X = Spd*	*X = PEI*
X	slight inhibition	-	81	77	77	62	86	62
X-propionate	inhibition in the case of spm	-	38	90	90	66	87	104
X-caproate	inhibition	-	70	69	60	53	117	53
X-lipoate	inhibition in the case of spd and PEI	-	100	23	57	80	55	54
**EcA alone**	cytostatic	-	67	67	67	58	58	58
EcA + X	mostly cytostatic	Synergy (except for spermine)	120 (97)	58 (45)	40 (31)	106 (66)	45 (39)	58 (36)
EcA + X-propionate		Synergy (except for spermine)	103 (39)	44 (40)	50 (45)	100 (66)	44 (38)	36 (37)
EcA + X-caproate		Addition	57 (40)	119 (82)	52 (31)	138 (73)	34 (40)	70 (37)
EcA + X-lipoate		Mainly Inhibition	60 (60)	283 (65)	28 (16)	61 (49)	60 (33)	69 (37)
**EcA-spm alone**	cytostatic	-	58	58	58	50	50	50
EcA-spm + X	less efficient cytostatics	Addition for A549 Inhibition for K562	46 (37)	40 (31)	25 (19)	116 (72)	51 (44)	71 (44)
EcA-spm + X-propionate		Inhibition for A549, Mainly Inhibition for K562	60 (42)	64 (44)	73 (44)	106 (56)	35 (41)	77 (41)
EcA-spm + X-caproate		Addition for A549 Mainly Inhibition for K562	45 (30)	88 (59)	28 (19)	134 (78)	174 (101)	48 (28)
EcA-spm + X-lipoate		Inhibition or indifferent	108 (42)	115 (46)	58 (26)	70 (46)	71 (27)	189 (70)

* The error of determination did not exceed 15%.

## Data Availability

The data presented in this study are available in the main text and Supplementary Materials.

## Supplementary Materials

The following supporting information can be downloaded at: https://www.mdpi.com/article/10.3390/ijms26146749/s1.

## Abbreviations

The following abbreviations are used in this manuscript:

CD	Circular dichroism
CLSM	confocal laser scanning microscopy
EcA	*Escherichia coli* L-asparaginase
EwA	*Erwinia carotowora* L-asparaginase
FTIR	Fourier-transform infrared
L-ASNase	L-asparaginase
PEI	Polyethyleneimine
Put	Putrescine
RrA	*Rhodospirillum rubrum* L-asparaginase
Spd	Spermidine
Spm	Spermine
TNBS	Trinitrobenzene sulfonic acid
