# Supplementary material for: Receptor-Mediated Internalization of L-Asparaginase into Tumor Cells Is Suppressed by Polyamines
Source: Int J Mol Sci. 2025 Jul 14;26(14):6749. doi: 10.3390/ijms26146749 (PMC12295751; doi:10.3390/ijms26146749)

Article

Receptor-mediated Internalization of L-Asparaginase into Tumor Cells is suppressed by polyamines

Igor D. Zlotnikov [1], Alexander A. Ezhov [2] and Elena V. Kudryashova [1,*]

[1] Faculty of Chemistry, Lomonosov Moscow State University, Leninskie Gory, 1/3, 119991 Moscow, Russia; zlotnikovid@my.msu.ru (I.D.Z.);

[2] Faculty of Physics, Lomonosov Moscow State University, Leninskie Gory, 1/2, 119991 Moscow, Russia; alexander-ezhov@yandex.ru

* Correspondence: helenakoudriachova@yandex.ru

Table S1. The receptors of the polyamine transportation system or enzymes related to the metabolism of amines and the prospects of their modulation in cancer therapy.

Receptor of PTS/ Target	Function	Expression in Cancer vs. Normal Cells	Role in Cancer Treatment (Activators/Inhibitors)	Specific examples	References
ATP13A4	Transport of various polyamines, including spermidine and spermine	Increased expression in breast cancer cells MCF7	Inhibition of receptors (e.g., by AMXT-1501 – derivative of spermine and decanoic acid) may be a therapeutic target in cancer	Breast cancer	[1,2]
ATP13A3		Is involved in polyamine transport in pancreatic cancer and neuroblastoma cells		Pancreatic cancer, neuroblastoma	[3–5]
SLC3A2/SLC7A5 (LAT1)	Neutral amino acid transporter	Often overexpressed in many types of cancer	Can facilitate the transport of some polyamines, particularly those with structural similarities to large neutral amino acids	Colorectal cancer, breast cancer, neuroblastoma, pancreatic cancer, head and neck cancer	[6,7]
Glypican-1 (GPC-1)	Driver of cancer cell proliferation and metastasis, exerting its influence through the regulation of angiogenesis processes.	GPC1 exhibits an aberrant expression in tumor tissues, where it is implicated in the pathogenesis of cancer	May indirectly participate in receptor-mediated polyamine endocytosis	Antibody–drug conjugates, CAR-T cell therapy, radiotherapy, bispecific T cell engagers, and immunotoxins	[8–10]
SLC18B1 (Vesicular monoamine transporter 2, VMAT2)	Vesicular storage of monoamines (serotonin, dopamine)	Proposed as a potential polyamine transporter	Involved in polyamine storage and release	The absence of Slc18b1 results in a decrease in the level of polyamines in neurons	[11,12]
SAT1 (Spermidine / spermine N1-acetyltransferase 1)	Inside the cells, SAT1 catalyzes the acetylation of spermidine and spermine. It shows relatively similar activity towards both spermidine and spermine.	Expression can be increased or decreased depending on the type of cancer; participates in polyamine catabolism	Inhibition of SAT1 can increase sensitivity to radio and chemotherapy; activation of SAT1 can promote glutamine metabolism and create vulnerability in lung cancer cells	Glioblastoma, melanoma, breast cancer, neuroblastoma, lung cancer, prostate cancer, colorectal cancer, low-grade glioma	[13–15]

Receptor of PTS/ Target	Function	Expression in Cancer vs. Normal Cells	Role in Cancer Treatment (Activators/Inhibitors)	Specific examples	References
SMOX (Spermine oxidase)	Inside the cells, SMOX oxidizes spermine to produce spermidine and hydrogen peroxide. It is highly specific to spermine and will not act on other polyamines.	Expression is often elevated in cancer cells	Inhibition of SMOX can be a therapeutic strategy in cancer, neurodegenerative diseases, and inflammation; induction of SMOX can be part of an anti-cancer strategy, leading to the formation of ROS	Stomach cancer, colorectal cancer, prostate cancer, breast cancer	[16–19]

References

1. Sarah, V.V.; Dolores, I.; Kristina, S.; Justin, S.; Sivadasan, B.D.; Elke, A.; Hanne, D.; Nina, S.; Chris, V.D.H.; Joris, V.A.; et al. *Astrocytic Polyamine Transport by ATP13A4 Tunes Excitatory Synaptic Transmission*; 2025; Vol. 4; ISBN 0000000157995.
2. van Veen, S.; Kourti, A.; Ausloos, E.; Van Asselberghs, J.; Van den Haute, C.; Baekelandt, V.; Eggermont, J.; Vangheluwe, P. ATP13A4 Upregulation Drives the Elevated Polyamine Transport System in the Breast Cancer Cell Line MCF7. *Biomolecules* **2023**, *13*, doi:10.3390/biom13060918.
3. Liu, B.; Azfar, M.; Legchenko, E.; West, J.A.; Martin, S.; Van Den Haute, C.; Baekelandt, V.; Wharton, J.; Howard, L.; Wilkins, M.R.; et al. ATP13A3 Variants Promote Pulmonary Arterial Hypertension by Disrupting Polyamine Transport. *Cardiovasc. Res.* **2024**, *120*, 756–768, doi:10.1093/cvr/cvae068.
4. Hamouda, N.N.; van den Haute, C.; Vanhoutte, R.; Sannerud, R.; Azfar, M.; Mayer, R.; Calabuig, Á.C.; Swinnen, J. V.; Agostinis, P.; Baekelandt, V.; et al. ATP13A3 Is a Major Component of the Enigmatic Mammalian Polyamine Transport System. *J. Biol. Chem.* **2021**, *296*, 100182, doi:10.1074/jbc.RA120.013908.
5. Azfar, M.; Gao, W.; Van den Haute, C.; Xiao, L.; Karsa, M.; Pandher, R.; Ronca, E.; Bongers, A.; Karsa, A.; Spurling, D.; et al. The Polyamine Transporter ATP13A3 Mediates DFMO-Induced Polyamine Uptake in Neuroblastoma 2024.
6. Zanatta, J.M.; Acuña, S.M.; Bento, C.D.A.; Stolf, B.S.; Muxel, S.M. Polyamines Shift Expression of Macrophage L-Arginine Metabolism Related-Genes during Leishmania Amazonensis Infection 2022, *2*.
7. Nachef, M.; Ali, A.K.; Almutairi, S.M.; Lee, S.H. Targeting SLC1A5 and SLC3A2/SLC7A5 as a Potential Strategy to Strengthen Anti-Tumor Immunity in the Tumor Microenvironment. *Front. Immunol.* **2021**, *12*, 1–11, doi:10.3389/fimmu.2021.624324.
8. Belting, M.; Mani, K.; Jönsson, M.; Cheng, F.; Sandgren, S.; Jonsson, S.; Ding, K.; Delcros, J.G.; Fransson, L.Å. Glypican-1 Is a Vehicle for Polyamine Uptake in Mammalian Cells: A Pivotal Role for Nitrosothiol-Derived Nitric Oxide. *J. Biol. Chem.* **2003**, *278*, 47181–47189, doi:10.1074/jbc.M308325200.
9. Cheng, F.; Fransson, L.-Å.; Mani, K. Common Traffic Routes for Imported Spermine and Endosomal Glypican-1-Derived Heparan Sulfate in Fibroblasts. *Exp. Cell Res.* **2018**, *364*, 133–142, doi:10.1016/j.yexcr.2018.01.029.
10. Cheng, F.; Mani, K.; van den Born, J.; Ding, K.; Belting, M.; Fransson, L.-Å. Nitric Oxide-Dependent Processing of Heparan Sulfate in Recycling S-Nitrosylated Glypican-1 Takes Place in Caveolin-1-Containing Endosomes. *J. Biol. Chem.* **2002**, *277*, 44431–44439, doi:10.1074/jbc.M205241200.
11. Fredriksson, R.; Sreedharan, S.; Nordenankar, K.; Alsiö, J.; Lindberg, F.A.; Hutchinson, A.; Eriksson, A.; Roshanbin, S.; Ciuculete, D.M.; Klockars, A.; et al. The Polyamine Transporter Slc18b1(VPAT) Is Important for Both Short and Long Time Memory and for Regulation of Polyamine Content in the Brain. *PLoS Genet.* **2019**, *15*, 1–30, doi:10.1371/journal.pgen.1008455.

12. Pochini, L. Involvement of Mammalian SoLute Carriers (SLC) in the Traffic of Polyamines. *Front. Mol. Biosci.* **2024**, *11*, 1–8, doi:10.3389/fmolb.2024.1452184.
13. Han, X.; Wang, D.; Yang, L.; Wang, N.; Shen, J.; Wang, J.; Zhang, L.; Chen, L.; Gao, S.; Zong, W.-X.; et al. Activation of Polyamine Catabolism Promotes Glutamine Metabolism and Creates a Targetable Vulnerability in Lung Cancer. *Proc. Natl. Acad. Sci.* **2024**, *121*, doi:10.1073/pnas.2319429121.
14. Ou, Y.; Wang, S.-J.; Li, D.; Chu, B.; Gu, W. Activation of SAT1 Engages Polyamine Metabolism with P53-Mediated Ferroptotic Responses. *Proc. Natl. Acad. Sci.* **2016**, *113*, doi:10.1073/pnas.1607152113.
15. Liu, T.-A.; Stewart, T.M.; Casero, R.A. The Synergistic Benefit of Combination Strategies Targeting Tumor Cell Polyamine Homeostasis. *Int. J. Mol. Sci.* **2024**, *25*, 8173, doi:10.3390/ijms25158173.
16. Cervelli, M.; Amendola, R.; Polticelli, F.; Mariottini, P. Spermine Oxidase: Ten Years After. *Amino Acids* **2012**, *42*, 441–450, doi:10.1007/s00726-011-1014-z.
17. Ohkubo, S.; Mancinelli, R.; Miglietta, S.; Cona, A.; Angelini, R.; Canettieri, G.; Spandidos, D.A.; Gaudio, E.; Agostinelli, E. Maize Polyamine Oxidase in the Presence of Spermine/Spermidine Induces the Apoptosis of LoVo Human Colon Adenocarcinoma Cells. *Int. J. Oncol.* **2019**, *54*, 2080–2094, doi:10.3892/ijo.2019.4780.
18. Thomas, T.; Thomas, T.J. Polyamine Metabolism and Cancer. *J. Cell. Mol. Med.* **2003**, *7*, 113–126, doi:10.1111/j.1582-4934.2003.tb00210.x.
19. Thomas, T.J.; Thomas, T. Cellular and Animal Model Studies on the Growth Inhibitory Effects of Polyamine Analogues on Breast Cancer. *Med. Sci. (Basel, Switzerland)* **2018**, *6*, doi:10.3390/medsci6010024.

Table S2. Number of titrated amino groups per L-ASNase tetramer.

	Native	spm	spm	spm	spm	spd	spd	spd	spd	put	put	put	put	PEI	PEI	PEI	PEI
RrA	28	30	33	37	53	30	32	37	51	30	33	36	50	31	35	55	88
EwA	80	84	88	97	122	84	89	96	116	84	88	94	115	86	92	119	148
EcA	88	91	94	103	126	92	96	105	127	92	95	106	128	96	102	126	151

Figure S1. Three-dimensional structural representation of RrA conjugated with spermine.

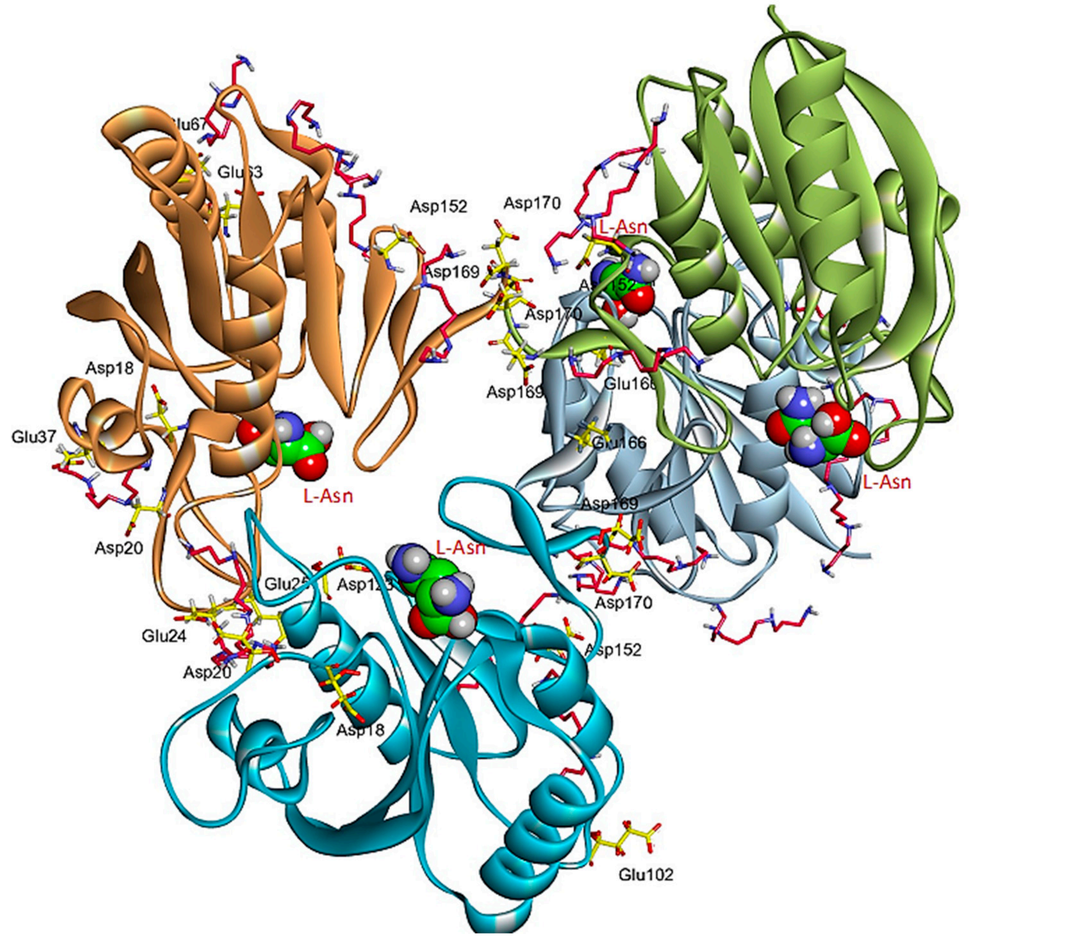

Figure S2. A schematic representation of the process for the independent production of fluorescently labelled asparaginase or its conjugates, as well as polyamines.

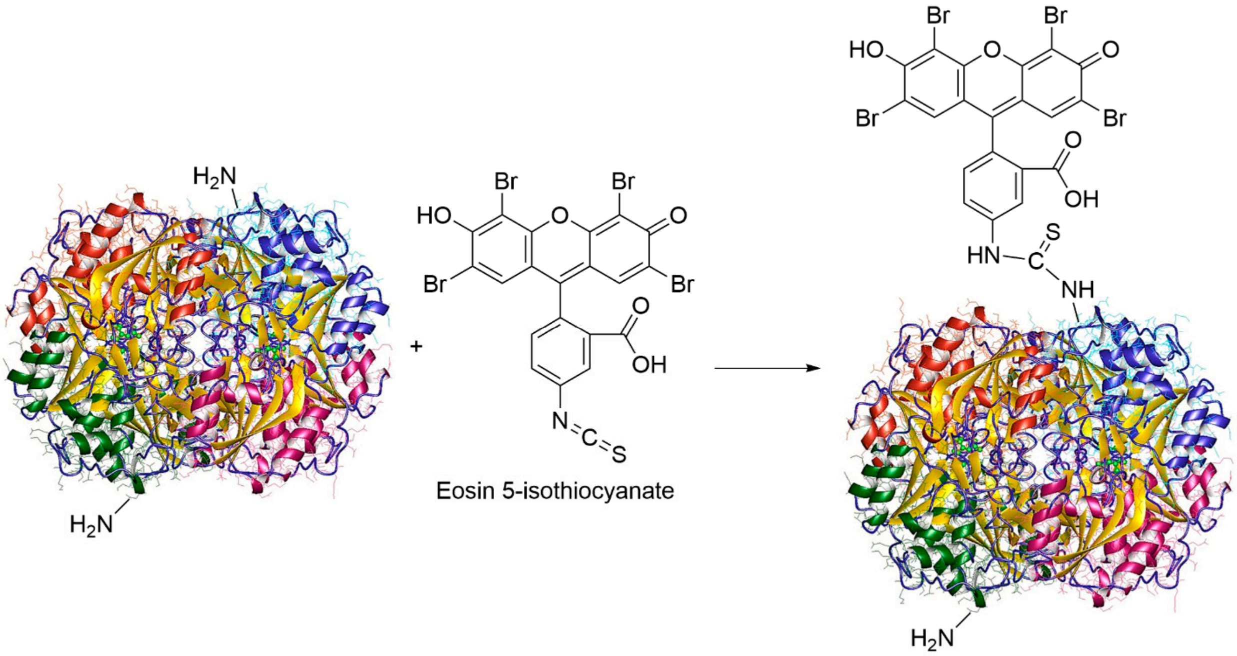

Supplement: Supplementary file 1 [file ijms-26-06749-s001.zip › ijms-3727227-supplementary.pdf]
